# 3D chromatin structure remodelling in cancer

**DOI:** 10.1186/s12964-026-03097-3

**Published:** 2026-07-27

**Authors:** Yang Liu, Minghua Wu

**Affiliations:** 1 Department of Scientific Research and Teaching, Hunan Aerospace Hospital, Hunan Normal University, Changsha, Hunan 410205 China; 2 The Key Laboratory of Carcinogenesis of the Chinese Ministry of Health, The Key Laboratory of Carcinogenesis and Cancer Invasion of the Chinese Ministry of Education, Cancer Research Institute, Central South University, Changsha, Hunan 410078 China; 3 FuRong Laboratory, Changsha, Hunan 410078 China; 4 School of Basic Medical Sciences, Hunan Normal University Health Science Center, Hunan Normal University, Changsha, Hunan 410013 China; 5 Xiangya School of Public Health, Central South University, Changsha, Hunan 410013 China

**Keywords:** 3D chromatin structure, 3D genomics, Tumor

## Abstract

Three-dimensional (3D) chromatin structure is crucial for gene regulation and cellular identity, with alterations in this structure being closely associated with tumor development. Although the research of tumor genomics has made significant progress in the past decade, the mechanism of 3D chromatin structure in tumorigenesis is not completely clear. Recent studies have shown that the distortion of chromatin spatial conformation has become one of the core factors driving normal cell carcinogenesis by changing the gene regulatory network. Abnormal high-order chromatin organization leads to abnormal activation of oncogenes or silencing of tumor suppressor genes. This 3D chromatin disorder has been confirmed to be directly related to the phenotypic plasticity of a variety of tumors. Therefore, the dynamic changes of 3D chromatin structure play a key role in the occurrence and development of tumors, especially for malignant tumors with difficult diagnosis and lack of treatment. This article provides an overview of hierarchical alterations in cancer 3D chromatin structure, the mechanisms governing these changes, recent advances in detection technologies, and emerging therapeutic strategies. The unique 3D chromatin variation pattern in cancers may become a new biomarker and provide a theoretical basis for epigenetic therapy targeting chromatin spatial organization. In the future, integrating multi-omics data to develop specific drugs for key nodes of the 3D chromatin and establishing an early diagnosis system based on spatial chromatin characteristics will become an important direction to break through the bottleneck of cancer treatment.

## Background

In the relatively narrow and precise nucleus space, chromatin exists in a highly complex and intricate folding manner. In the long course of biological evolution, the genome of eukaryotes is relatively conserved as a whole, and many core elements, such as key gene sequences and basic structures, are stably inherited among different species, providing a basic guarantee for eukaryotes to maintain basic biological characteristics and stable operation of life activities. However, eukaryotic genomes are highly plastic at the spatial level. Its spatial structure is not fixed, but can be flexibly adjusted and changed according to the biological environment, cell physiological state and development stage [1]. The dynamic changes of chromatin structure in nucleus make the spatial layout of genome and gene accessibility more flexible, which can better adapt to the needs of gene expression regulation in different situations, and provide the material basis and regulation mechanism for eukaryotes to show their diverse adaptability and evolutionary potential.

Modern three-dimensional (3D) genome research, based on spatial interaction hierarchy, divides the higher-order 3D structure of chromatin into several different-level functional modules, including A/B compartments, topologically associating domains (TADs), and chromatin loops [2, 3]. Compartment A is usually associated with regions with dense genes and active transcription, while compartment B is associated with regions with sparse genes and inactive transcription. TADs are the structural units of chromatin interaction, and their internal chromatin interaction frequency is high, which guide and promote chromatin interactions [4]. TADs are formed by interactions mediated by CTCF and other structural proteins. CTCF and cohesin are key proteins that regulate the 3D genome structure [5]. Chromatin loops are crucial for transcriptional regulation and expression of genes. They regulate the interaction between genes or between genes and their regulatory factors, thereby affecting gene expression [6–8]. Abnormal chromatin interactions mediate the occurrence of diseases. Therefore, accurate detection of chromatin interactions is of great significance for the study of gene transcription and expression, disease diagnosis, treatment and drug targets.

Tumor is one of the major diseases that seriously threaten human health worldwide, and its morbidity and mortality remain high. Malignant tumors have the characteristics of self-sufficient growth signal, insensitive to growth inhibition signal, unlimited replication potential, escape from apoptosis, tissue invasion and metastasis, and continuous angiogenesis, which promote cells from normal state to tumor growth state. Epigenetic regulation is the regulation mechanism of genetic changes in gene expression or cell characteristics without changing the DNA sequence, including 3D chromatin structure, histone modification, DNA methylation and so on. Epigenetic reprogramming has been identified as one of the hallmark features of cancer [9]. The epigenetic changes of various tumors have been gradually analyzed, but the mechanism of the 3D structure of chromatin in tumors is not completely clear.

In this review, we summarize hierarchical alterations in cancer 3D chromatin structure, discuss their multifaceted regulatory mechanisms, highlight advances in detection technologies, and review current therapeutic strategies targeting 3D genome, aiming to provide insights into tumor pathogenesis and inform precision oncology.

## Extensive 3D chromatin structure alterations in cancer

**Fig. 1:**
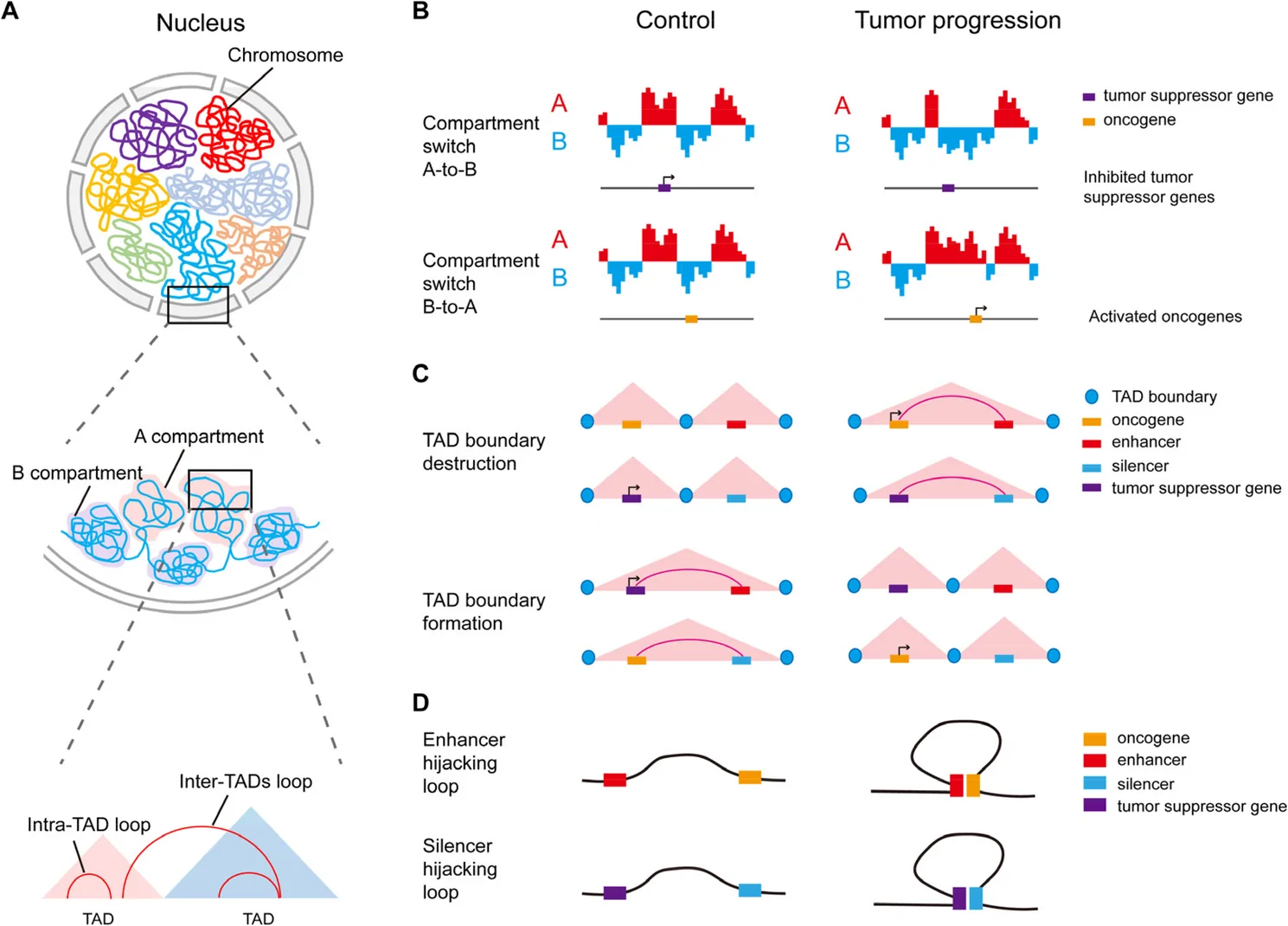
Potential mechanism of 3D chromatin structure regulating tumor progression. **A** Chromatin folds and forms 3D structures at different hierarchical levels within the nucleus of the cell. **B** Schematic of A/B compartment switching in tumor progression. **C** Dysregulation of TAD boundaries in tumor progression. **D** Aberrant chromatin loops drive tumor progression, including enhancer hijacking loops and silencer hijacking loops

The hierarchical structure of the nuclear genome is composed of chromosomes, compartments, TADs, and chromatin loops (Fig. 1A). The potential mechanisms by which 3D chromatin structure regulates tumor progression are also reflected in changes at different structural levels.

### Compartment alterations in cancer

The 3D chromatin structure of tumors undergoes extensive remodeling at the compartment level. Compared with normal cells, the total incidence of A/B compartment switch in pancreatic ductal adenocarcinoma cell line BxPC3 was 24.8% (15.3% from compartment A to compartment B and 9.5% from compartment B to compartment A). The total incidence of A/B compartment switch in pancreatic ductal adenocarcinoma cell line PANC1 was 24.1% (16.2% from compartment A to compartment B and 7.9% from compartment B to compartment A). PHLDA1 is a gene that occurs in the region of compartment B to compartment A transition on chromosome 12 in cancer cells, and its expression is significantly up-regulated in cancer cell lines, which is significantly associated with poor prognosis of pancreatic cancer [10]. On average, 3.2% of acute myeloid leukemia (AML) patients had A-to-B compartment switch and 2.9% of AML patients had B-to-A compartment switch [11]. A/B compartment switch in bone marrow plasma cells of patients with multiple myeloma (408 bins were switching from compartment B to compartment A and 321 bins from compartment A to compartment B) activates the expression of development related transcription factor genes and silences the expression of cell adhesion molecule genes n^12^rrangement of chromatin compartments o ADDIN EN.CITE about 5.2% of the gene regions changed from inhibitory B compartment to activated A compartment, which was significantly correlated with the up-regulation of gene expression [12]. Compared to the chromatin structure of normal brain tissue, approximately 5.78% of the genomic regions in glioblastoma (GBM) transition from an open chromatin state to a condensed chromatin region (A-to-B transition), and 6.78% transition from a condensed chromatin state to an open region (B-to-A transition). Gene expression is closely correlated with these compartmental changes. Taking the CHI3L2 gene as an example, this gene is closely associated with poor prognosis and immune infiltration in glioma patients. Its expression level is low in normal tissue samples but significantly elevated in GBM samples. Conversely, the tumor suppressor genes CAVIN2 and TMEFF2 are downregulated in GBM samples [13]. During the progression of breast tumors, 46.8% normal tissues A switch to ER+ breast tumors B and 32.8% normal tissues B switch to ER+ breast tumors A [14]. These results indicate that significant differences exist in the direction of compartment transition in different cancer types.

Compared to the changes in the 3D chromatin characteristics of primary cancer cells, some metastatic cancer cells exhibit more significant changes. The compartment switching between metastasis and normal/primary pancreatic cancer cells was more substantial (26.70% in metastasis vs. normal and 30.75% in metastasis vs. Primary), than the compartment switching between normal pancreatic ductal and primary cancer cells (16.17%). In some cases, regions of the genome that are in B compartment in normal or primary cancer cells switch to A compartment in metastatic cancer cells. The genes in these regions are enriched for functions related to the Ras/small GTPase pathway, suggesting that A/B compartment reorganization may facilitate the metastasis of pancreatic cancer [15]. For colorectal carcinoma, the proportion of compartments that switched A/B status between normal and tumor samples ranged from 4.73% to 18.99% and the proportion between primary and metastatic tumors ranged from 6.35% to 21.62% in individual patients. A significantly higher compartment stability score was observed in patients without metastasis [16]. The stability of cellular compartments in patients with these two types of metastatic tumors is lower than that of normal cells and primary cancer cells, making them more prone to A/B switching.

3D chromatin characteristics of drug-treated recurrent tumors and drug-resistant tumors also exhibit substantial A/B transitions. In the transition from primary tumors to tamoxifen-treated recurrent breast tumors, 15.3% of chromatin regions switch from A compartment to B compartment and 58.3% of chromatin regions switch from B compartment to A compartment [14]. In oxaliplatin-resistant colorectal cancer, it was found that 19.33% of the genome compartment properties changed during drug resistance, including 9.18% from A compartment to B compartment, and 10.15% from B compartment to A compartment [17]. Interestingly, the B-A flipping transition is more dominant in both drug-treated recurrent tumors and drug-resistant tumors mentioned above.

In summary, during the development and progression of tumors, there is a significant transition between compartment A and compartment B. The conversion of A/B compartment was significantly correlated with the change of gene expression. The gene expression level in the switch from A compartment to B compartment tended to decrease, while the gene expression level in the switch from B compartment to A compartment tended to increase. Some oncogenes that are originally located in the B compartment may be mistakenly transferred to the A compartment, thereby being abnormally activated. Conversely, some tumor suppressor genes that are originally located in the A compartment may be mistakenly transferred to the B compartment, thereby being abnormally inhibited (Fig. 1B).

### TAD alterations in cancer

Cancer cells display widespread remodeling of TADs. For example, in pancreatic cancer, 18.46% of the cell-specific contact domain boundaries are present in normal cells HPDE6C7, 19.24% in cancer cells BxPC3, and 15.12% in cancer cells PANC1. Compared to normal cells, pancreatic cancer cells tend to exhibit a trend of boundary disappearance in contact domain boundaries. There is diversity in the changes of contact domains across different chromosomes. Compared to stable contact domains, cancer-specific contact domains tend to be associated with upregulated gene expression. Among the shared boundaries of cancer-specific contact domains, approximately 42% of the gene expression levels show significant differences between the two pancreatic cancer cell lines compared to normal cells, including some key genes (such as TP53, SMAD3, PDGFA, etc.). Differentially expressed genes in cancer-specific contact domains are involved in multiple key pathways, including cancer promotion, cell differentiation, cell adhesion, cell movement, and cell migration, and alterations in these pathways may be closely related to the occurrence and development of cancer [10]. Furthermore, studies have found that the contact domain size of metastatic pancreatic cancer cells is significantly smaller than that of normal and primary cancer cells, while the number of contact domain boundaries has increased, indicating the formation of new contact domain boundaries during metastasis [15]. TADs are highly heterogeneous in breast tumors, and studies have found that the TAD size of significantly variable TADs is larger than that of moderately variable TADs and inter-tumor conserved TADs [14]. In the oxaliplatin-resistant group of colorectal cancer, 42.45% of TAD boundaries are weakened, and 31.38% are strengthened [17]. The multiple myeloma genome presents more but smaller TADs. Multiple myeloma has 19 specific TAD boundaries involving 43 genes such as FBXW11 and TNFRSF1B, significantly enriched in the Wnt pathway, HIV-1 infection, and cytokine receptor interaction pathways. Among them, changes in the TAD boundary where NFATC1 is located may affect the progression of myeloma [18]. There are significant differences in TAD structure between diffuse large B-cell lymphoma (DLBCL) and normal B cells. Studies have found that most changes in TAD structure are specific acquisitions of newly formed TADs in patients, while the loss of TADs present in normal B cells in patients is relatively rare. One significant acquired TAD structure observed includes the cancer-related gene TP63 and its regulatory gene TPRG, suggesting that they may have a co-regulatory relationship [19]. During the disease progression from chronic lymphocytic leukemia to DLBCL, the interactions within TADs increase, while the interactions between TADs decrease, indicating that the TAD structure becomes more closed [20].

In brief, TAD restructuring alters local chromatin accessibility and cis-regulatory interactions by weakening or strengthening domain boundaries. Boundary disruption allows regulatory elements to cross into adjacent TADs: enhancers may aberrantly activate oncogenes, while silencers may suppress tumor suppressor genes, promoting tumorigenesis. Conversely, de novo TAD boundary formation can isolate genes from their normal regulatory elements, sequestering a tumor suppressor from its enhancers or an oncogene from its silencer and leading to aberrant gene silencing or activation (Fig. 1C).

### Chromatin loop alterations in cancer

Chromatin loops, as the fundamental functional units of 3D genomic structure, precisely regulate gene expression by mediating interactions between regulatory elements and promoters. Their abnormal remodeling in tumor cells has become an important mechanism driving carcinogenesis and epigenetic dysregulation.

The number of chromatin loops in pancreatic cancer cells significantly increases, and most of the cancer-specific chromatin loops are associated with active enhancers. Cancer-specific enhancer-promoter loops are significantly correlated with the significant upregulation of gene expression, including genes such as TP63, LAMA3, CD58, and MET. The functions of genes significantly upregulated in pancreatic cancer metastasis-specific enhancer-looped genes are enriched in cell migration and angiogenesis. Metastasis-specific enhancers form chromatin loops with the promoters of key genes such as LIPC and KLF5, thereby promoting pancreatic cancer metastasis [10, 15, 21]. In metastatic castration-resistant prostate cancer, multiple enhancer-promoter interactions exist in key oncogene regions (FOXA1, MYC, and AR), and the correlation between enhancers and gene expression weakens with increasing distance [22].

Chromatin loops exhibit high heterogeneity in breast tumors. Acquired looping genes such as CA2, NUBPL, and ATP2B2 suggest their potential role in drug resistance. Among them, research has confirmed the crucial role of the CA2 loop in tamoxifen resistance in breast tumors [14].

The promoter-enhancer loops in the early stages of colorectal cancer gradually lose their interaction as carcinogenesis progresses, correlating with the shift from a redundant to a rate-limiting state of gene transcription, which serves as the fundamental mechanism underlying cancer gene dysregulation [23]. The enhancer-promoter loop interaction of the key gene SPP1 continuously increases during the development and metastasis of colorectal cancer [16]. The novel enhancer-promoter loop in the LEF1 gene may be associated with oxaliplatin resistance in colorectal cancer [17].

In AML-specific chromatin loops, 42.4% of the specific loops are formed between gene promoters and enhancers, while 11.2% are formed between promoters and silencers. Specific promoter-enhancer loops contain many known proto-oncogenes (such as MYCN, WT1, ERG, MEIS1, and RUNX1). Distal silencers inhibit the expression of their target genes (such as IKZF2 and RTTN) by forming inhibitory chromatin loops. The expression level of genes anchored by AML-specific promoter-enhancer loops is higher than that of genes anchored by promoter-silencer loops [11]. In T-cell acute lymphoblastic leukemia (T-ALL), oncogenes typically alter gene expression by hijacking enhancers, including the hijacking of T-cell receptor enhancers to activate LMO1, LMO2, NKX2-1, HOXA9, TLX1, and TLX3, and the hijacking of distal enhancers to activate the expression of HOXA13. In Notch inhibitor-resistant cells, the LEF1 and IKZF2 enhancer-promoter hubs undergo recombination, activating resistance-related transcriptional programs [24–27].

In the chromatin loop reprogramming of multiple myeloma, promoter-enhancer interaction loops account for the highest proportion. In mantle cell lymphoma resistant to ibrutinib, the formation of enhancer-promoter hubs leads to upregulation of drug resistance-related genes such as BCL2L1, TPX2, and DNMT3B [18, 27].

The Group 3 molecular subgroup of medulloblastoma forms a unique enhancer-promoter chromatin loop at the LIN28B gene, leading to specific transcriptional upregulation of this subgroup [28, 29]. The oncogenes GFI1 and GFI1B are repeatedly activated through enhancer hijacking in the Group 3 and Group 4 molecular subgroups of medulloblastoma [30].

In summary, the abnormal alterations in tumor-associated chromatin loop structures not only profoundly affect the transcriptional regulatory network of oncogene activation and tumor suppressor gene silencing, but also provide new epigenetic markers and research directions for tumor molecular typing, prognostic assessment, and the development of targeted intervention strategies (Fig. 1D).

## Determinants of 3D chromatin structure in cancer

The regulation of 3D chromatin structure in tumors is a complex network of multi-level and multi factor synergistic effects. There is a cross dialogue between different regulatory factors. These mechanisms together form the structural basis for the disruption of gene expression programs in tumor development, and provide new directions for targeted 3D genome therapy strategies.

### Structural variation

**Fig. 2:**
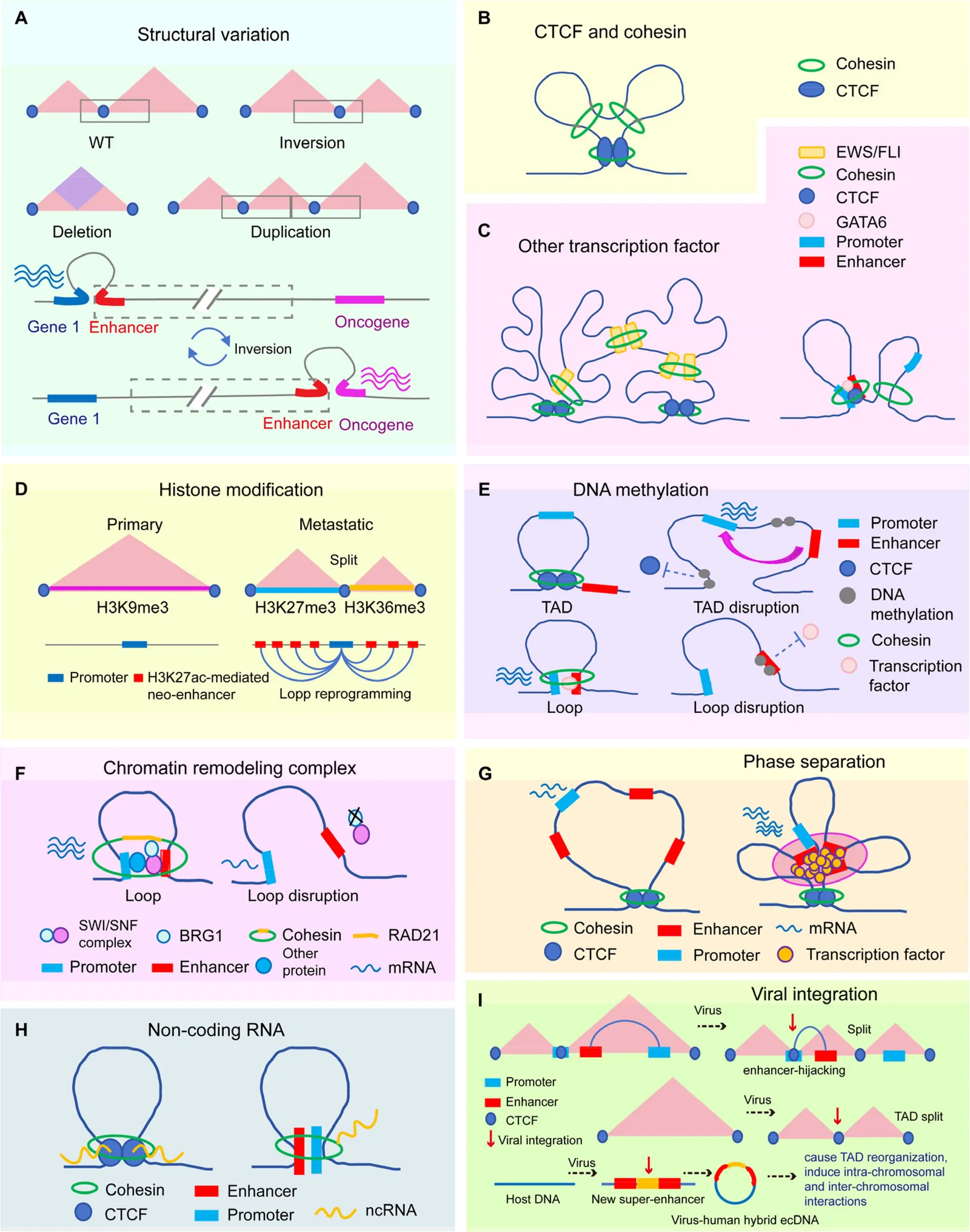
Determinants of 3D chromatin structure in cancer. **A** SVs alter TADs and ectopic loops. **B** CTCF and cohesin establish loops and insulation. **C** Transcription factors (e.g., EWS/FLI1, GATA6) regulate chromatin contacts. **D** Histone modifications modulate compaction and loop formation. **E** DNA methylation at architectural sites weakens TAD insulation and disrupts loop. **F** Chromatin remodeling complex controls higher-order structure. **G** Phase separation drives spatial reorganization of chromatin. **H** ncRNAs cooperate with CTCF/cohesin to shape 3D genome. **I** Viral integration disrupts host TADs, induces enhancer-hijacking and generates ecDNAs to regulate 3D chromatin structure. Collectively, genetic, epigenetic, and biophysical inputs converge to sculpt the cancer 3D genome

Structural variations (SVs), including deletions, inversions, duplications, and translocations, lead to changes in 3D chromatin structure and play a significant role in tumorigenesis and progression (Fig. 2A).

SVs mediate chromatin compartment reorganization and TAD rearrangement. In the chromatin translocation regions of colorectal cancer, A/B compartments undergo switching, and TAD hierarchies undergo remodeling. SV breakpoints tend to form new TAD boundaries and increase the TAD hierarchy of SV flanking genes [16]. The duplication of 7p11.2 in EGFR-amplified glioblastoma activates EGFR expression by forming new TADs and new enhancer-promoter interactions between LINC01446 and EGFR [31]. In T-ALL, minor deletions in the insulator region allow oncogenes to establish new connections with previously unrelated enhancers, such as the deletion of the TAD boundary between TAL1 and STIL, leading to overexpression of the TAL1 gene [32].

Copy number variations reshape the 3D chromatin structure of tumors. 39% of the differential loops in multiple myeloma are associated with copy number variation genes. Among them, GPR18 causes weakened chromatin interactions due to copy number deletion [18].

SVs induce enhancer hijacking and silencer hijacking events. In AML, hijacked enhancers play a role in cell growth, while hijacked silencers have the effect of regulating the downregulation of target gene expression [11]. Chromosomal translocation or inversion in AML leads to the relocation of the enhancer of GATA2 to the vicinity of the EVI1 gene, forming a enhancer-promoter loop, thereby activating EVI1 expression and inhibiting GATA2, promoting leukemia progression [33, 34]. Chromosomal translocation hijacks the BCL11B enhancer to activate the TLX3 gene, promoting the occurrence and development of leukemia [35]. Chromosomal translocation events in diffuse intrinsic pontine glioma lead to the formation of new chromatin loops between the A2M promoter and enhancer. This enhancer hijacking event results in high gene expression [36]. SVs in GBM can not only induce enhancer hijacking but also lead to the loss of enhancers for the same gene, both of which result in gene expression dysregulation [13]. SVs facilitate the interaction between the SNCAIP super-enhancer and the PRDM6 promoter, leading to the abnormal activation of the PRDM6 gene in Group 4 medulloblastoma [37].

What’s more, the 3D structure of the genome also has a potential impact on the formation of SVs. For instance, studies have shown that in metastatic castration-resistant prostate cancer, structural variants with both breakpoints located within the same TAD occur at a significantly higher frequency than expected by random chance [22].

In summary, structural variations remodel the 3D genome of tumors by mediating chromatin compartment reorganization, TAD rearrangement, copy number variations, enhancer hijacking events and silencer hijacking events, leading to gene expression dysregulation.

### CTCF and cohesin

CCCTC-binding factor (CTCF) and cohesin are key regulatory proteins of 3D chromatin structure (Fig. 2B). The cohesin complex consists of four structural subunits, SMC1A, SMC3, RAD21, and either STAG1 or STAG2, forming a ring around DNA. CTCF is the main boundary factor for cohesin ring extrusion. Abnormalities in CTCF and cohesin affect 3D genome organization. TAD activity in T-ALL are closely related to the CTCF binding changes of the active TAD boundary, and the stronger the insulation of CTCF, the stronger the TAD activity. The merging event of TAD is related to the absence of CTCT, and TAD merging directly leads to the interaction between MYC gene and distal super enhancer [38]. In childhood gamma delta T-ALL, inactivation of the cohesin subunit STAG2 leads to abnormal TAD structure, disrupting the expression of T cell differentiation related genes [39]. In Ewing sarcoma, the absence of STAG2 interferes with the EWS/FLI1 driven oncogenic program by altering the promoter-enhancer interaction [40].

### Other transcription factor

In addition to CTCF and cohesin, there are also other proteins involved in regulating the 3D chromatin structure of tumors (Fig. 2C). In B-cell lymphoma (BCL), the transcription factor Prdm1 may participate in TAD stability regulation by binding to TAD boundaries in a CTCF independent manner [12]. The transcription factor MYC mediates chromatin remodeling in prostate cancer by interacting with CTCF protein. MYC enhances the binding rate of CTCF at these sites and disrupt the enhancer-promoter loop of plasticity genes in the neuroendocrine lineage [41]. GATA6 protein physically interacts with CTCF to regulate the 3D genome tissue of colorectal cancer [42]. The fusion transcription factor EWS/FLI colocalizes with cohesin, jointly promoting the formation of chromatin loops and the establishment of TAD boundaries in Ewing sarcoma [43].

### Histone and histone modification

Histone mutations and histone modifying enzyme mutations lead to dysregulation of the 3D chromatin structure of tumors (Fig. 2D). The histone H1 is associated with higher-order chromatin compaction and regulates the balance between repressive and active chromatin domains [44–46]. Histone H1 mutations are highly frequent in BCL. The destruction of H1 function leads to significant structural remodeling of the genome. This decompaction state causes specific changes in the epigenetic state, mainly reflected in the gain of H3K36me2 and/or the loss of the inhibitory marker H3K27me3. Under normal conditions, H1 proteins isolate early developmental genes into structurally inaccessible genomic compartments. H1 mutation mainly drives malignant transformation through 3D genome reconstruction, which leads to epigenetic reprogramming and abnormal reactivation of developmental silent genes [45]. Compared with the H3 wild-type GBM cell line, the H3K27M mutant pediatric high-grade glioma has more TAD boundaries and longer loop distances [36]. EZH2 protein is the catalytic subunit of PRC2. Its classical function is to catalyze H3K27me3 modification, thereby inhibiting gene transcription [47]. Studies have shown that there is a functional interplay between TADs and epigenetic and transcriptional changes mediated by mutated EZH2, which affects the occurrence of BCL. This mutation abnormally increases the level of H3K27me3, thereby inhibiting genes transcription. These inhibited genes have higher consistency within TAD and regulate the interaction within TAD. This epigenetic and transcriptional reprogramming that occurs synergistically within TAD leads to the common inactivation of multiple tumor suppressors, enhancing cell proliferation and tumor growth [48]. During pancreatic cancer metastasis, reprogramming of histone modifications can be accompanied by reprogramming of compartments and contact domains and enhancer reprogramming can be accompanied by loop reprogramming [15].

### DNA methylation

Widespread DNA methylation changes are frequently documented in cancer, where they disrupt structural regulatory factor binding and promote pathogenic chromatin interactions (Fig. 2E). The binding of CTCF is sensitive to DNA methylation [49, 50]. DNA methylation at CTCF binding sites prevents CTCF binding, leading to disappearance of TAD boundaries and activation of oncogenes. Methylation of CTCF binding sites in IDH mutant gliomas weakens the binding of CTCF, and the loss of CTCF binding at TAD boundaries leads to abnormal interaction between enhancers and PDGFRA. Increased expression of PDGFRA promotes the development of gliomas [51]. DNA methylation dysregulation is associated with 3D genome recombination in AML. Hypomethylating agent treatment partially restored chromatin structure, including the reversal of compartment switching and the disruption of cancer-specific loops [11]. In endocrine-resistant breast cancer, hypermethylation at estrogen receptor (ER)-occupied enhancers coincides with diminished ER occupancy and the disruption of ER-mediated enhancer-promoter chromatin contacts, ultimately leading to reduced transcription of ER-target genes [52].

### Chromatin remodeling complex

Chromatin remodeling complex also participates in regulating 3D chromatin structure (Fig. 2F). ARID1A serves as a subunit of the SWI/SNF chromatin remodeling complex. In liver cancer, ARID1A deficiency triggers A/B compartment switching, TAD reorganization, and reduced chromatin looping. The ATPase BRG1 within the SWI/SNF complex physically associates with the cohesin subunit RAD21, while ARID1A deficiency markedly weakens the BRG1-RAD21 interaction. The conformational changes induced by ARID1A deficiency lead to dysregulation of genes such as PMP22 and GSC, which enhances the invasive potential of liver cancer cells [53]. In prostate cancer, degradation of SWI/SNF ATPase subunits dismantles looping interactions between super-enhancers and gene promoters, thereby abolishing the hyperactivated expression of the FOXA1, AR and MYC oncogenes [54].

### Phase separation

Liquid-liquid phase separation is a new mechanism of chromatin structure regulation discovered in recent years (Fig. 2G). Some proteins with intrinsically disordered regions form transcriptional condensates through liquid-liquid phase separation, thus remodeling chromatin structure. In leukemia, the NUP98-HOXA9 fusion protein is a typical example. This fusion protein retains the intrinsically disordered region of NUP98, which enables it to form liquid-liquid phase separation droplets. This promotes its extensive binding to chromatin, creating a ‘super-enhancer’-like structure, and activates the expression of a series of leukemia-related genes. Studies have also found that the chromatin loop structures induced by liquid-liquid phase separation do not rely on the conventional CTCF/cohesin mechanism. This indicates that liquid-liquid phase separation can independently drive the spatial reorganization of chromatin, thereby participating in tumorigenesis [55].

### Non-coding RNA

Non-coding RNA is involved in regulating the 3D genome structure of cancer (Fig. 2H). R-loop formation driven by HOTTIP lncRNA via binding to complementary sequences governs CTCF boundary dynamics and TAD topology at β-catenin and related target loci, ultimately fueling malignant transcription programs and the development of leukemia [56]. In colorectal cancer, CCAT1-L is transcribed from the upstream super-enhancer of MYC and promotes CTCF dependent chromatin loops at the MYC locus [57]. Estrogen-regulated eRNAs stabilize chromatin looping in breast cancer cells by interacting with cohesin complex proteins, including RAD21 and SMC3 [58].

### Viral integration

Viral integration reshapes the 3D chromatin structure of host tumors, thereby driving carcinogenesis (Fig. 2I). HPV integration can induce chromatin topology alterations by introducing novel CTCF binding sites in the host genome [59]. In cervical cancer, HPV integration splits one TAD into two smaller TADs and hijacks the enhancer from PEG3 to CCDC106, resulting in reduced PEG3 expression and elevated CCDC106 expression [60]. HPV integration at the LINC00393 site also induces TAD division, resulting in reduced expression of the tumor suppressor KLF12 [61]. HPV integration generates cellular super-enhancers that function as HPV-human hybrid ecDNAs to induce 3D genomic structural changes and regulate gene transcription [62].

## Technological advances in 3D genome analysis

**Fig. 3:**
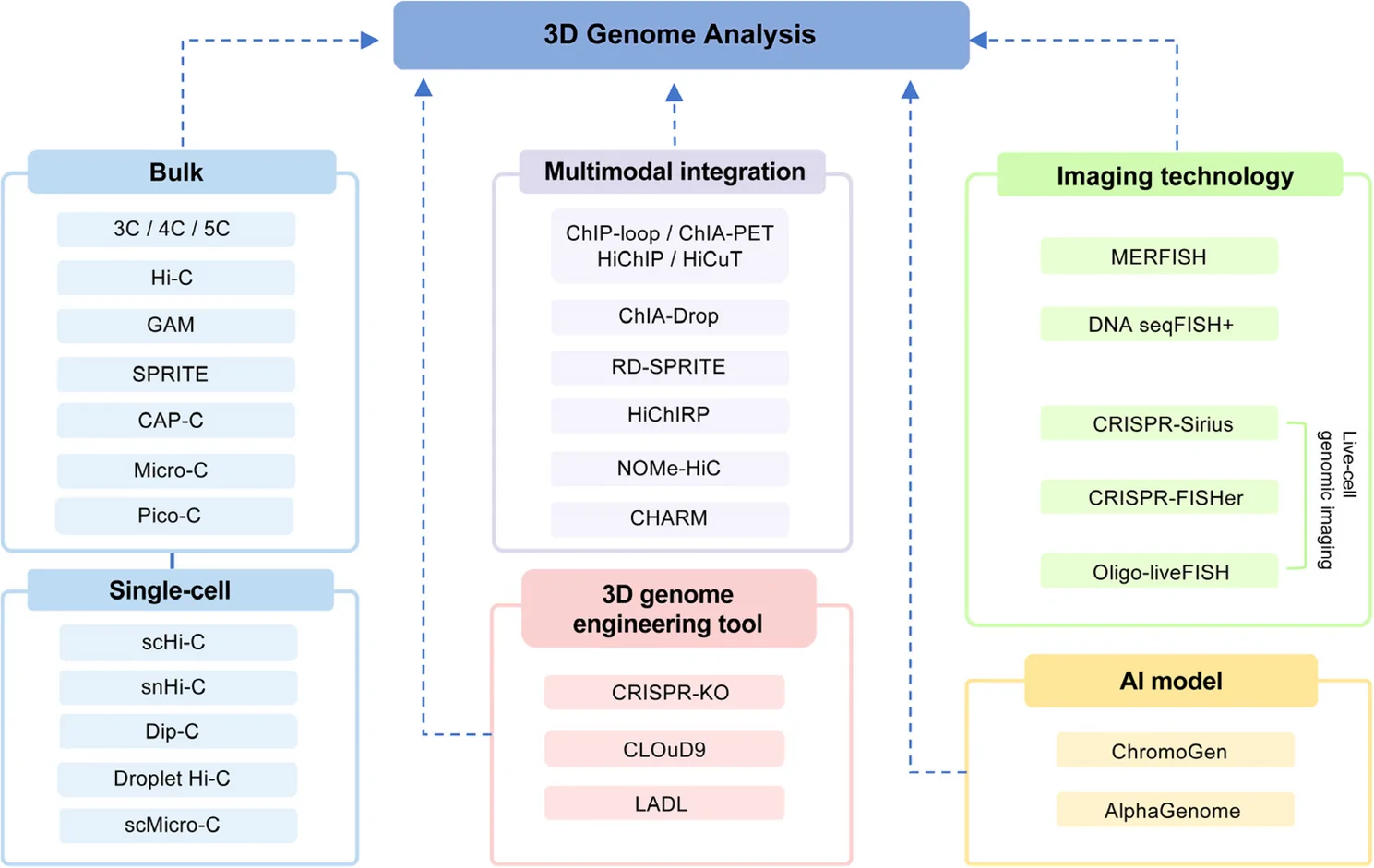
Technological advances in 3D genome analysis. Sequencing-based approaches for 3D chromatin structure are divided into bulk and single-cell approach. Multimodal integration technologies enable multi-dimensional dissection of gene regulatory mechanisms through simultaneous multi-omics profiling. Imaging technologies enable direct spatial mapping of chromatin loci in single cells. 3D genome engineering tools enable artificially manipulate chromatin structure and verify the changes in 3D structure. AI models enable efficient and large-scale prediction of 3D chromatin structure

In order to further study chromatin interactions in the 3D genome, various methods based on different strategies have emerged in the past few decades (Fig. 3). In 2002, Job Dekker and his team pioneered 3 C technology, which is mainly used to detect the spatial interaction between two specific sites in chromatin [63]. Based on 3 C technology, 4 C technology detects the interaction between a certain gene region and multiple or even all other regions, while 5 C technology detects the interaction between all loci within a specific region [64, 65]. In 2009, Job Dekker and his team achieved a breakthrough in spatial interaction detection at the whole genome level by developing Hi-C technology [3]. Hi-C obtains spatial interactions across the entire genome and has been widely used in the exploration of spatial structures in numerous species. GAM infers chromatin 3D spatial proximity by analyzing the co-occurrence frequencies of DNA fragments across ultrathin nuclear sections, bypassing the need for crosslinking and proximity ligation [66]. SPRITE enables genome-wide detection of higher-order interactions within the nucleus without proximity ligation [67]. CAP-C uses chemical crosslinkers to capture chromatin contacts, which avoids interference from DNA-bound proteins [68]. Micro-C technology is an ultra high resolution chromatin conformation capture technique that enhances resolution to the level of nucleosomes [69]. Pico-C is an improved method based on Micro-C that significantly reduces the initial cell count [70].

To capture chromatin conformational heterogeneity at the single-cell level, single-cell 3D genomics technology has emerged, achieving a paradigm shift from population averaging to individual resolution. In 2013, the Nagano team successfully pioneered single-cell Hi-C technology by using microscopy to separate cell nuclei [71]. SnHi-C technology achieves a tenfold increase in the number of single-cell chromatin interactions [72]. Dip-C achieves single-cell million level interaction detection [73]. Droplet Hi-C uses a commercial microfluidic device for high-throughput single-cell chromatin conformation analysis in droplets [74]. The scMicro-C technology supports high-resolution single-cell chromatin spatial structure detection [75].

To overcome the limitation of single-modal approaches that only provide chromatin spatial conformation information, multimodal integration technologies have enabled multi-dimensional dissection of gene regulatory mechanisms by simultaneously capturing 3D genome architecture, epigenetic modification states, transcriptional activity landscapes, RNA-chromatin associations, and protein-mediated DNA interactions. The technologies developed by multimodal integration of protein and 3D chromatin interactions include ChIP-loop, ChIA-PET, HiChIP, HiCuT, etc [76–79]. ChIP-loop technology combines the advantages of 3 C and ChIP-seq, specifically designed to detect interactions between two specific genes mediated by target proteins [76]. ChIA-PET and HiChIP technologies both bind Hi-C to ChIP-seq and detect all interactions mediated by the target protein [77, 78]. HiChIP technology has the advantages of higher sensitivity and lower sample requirements compared to ChIA-PET technology. HiCuT integrates Hi-C with CUT&Tag, offering a shorter experimental workflow, lower cell input demands, and reduced sequencing requirements [79]. ChIA-Drop technology combines ChIA-PET with microfluidic droplet technology to realize the analysis of chromatin interaction at the single molecular level [80]. RD-SPRITE generates a global map of 3D structures by simultaneously measuring RNA-RNA, RNA-DNA, and DNA-DNA contacts throughout the entire genome [81]. HiChIRP also enrichs RNA-related chromatin interactions [82]. MUSIC simultaneously analyzes multiple chromatin interactions, gene expression, and RNA-chromatin associations in single cells [83]. NOMe-HiC provides joint profiling of 3D genome, chromatin accessibility, genetic variant and DNA methylation in the same DNA molecule [84]. CHARM synchronously captures the 3D structure of the genome, chromatin accessibility, histone modifications, and transcriptome within the same cell [85].

Imaging techniques enable direct measurement of where chromatin loci are located in space within single cells. MERFISH is a method used for genome-scale chromatin tracking, which can simultaneously image genomic loci, nascent transcripts of genes, and landmark nuclear structures [86]. DNA seqFISH + is used to image and resolve the 3D spatial arrangement of massive numbers of DNA loci at the single-cell scale [87]. Live-cell multicolor genomic imaging technologies enable real-time tracking of specific chromatin dynamic structures in both space and time, including CRISPR-Sirius, CRISPR-FISHer and Oligo-liveFISH [88].

In order to artificially manipulate chromatin structure and verify the changes in 3D structure, researchers have also developed various CRISPR-based 3D genome engineering tools, such as CRISPR-KO, CLOuD9, LADL and BPCL [88].

Artificial intelligence (AI) models have achieved efficient and large-scale prediction of chromatin 3D conformation. The AI model ChromoGen leverages underlying DNA sequences and chromatin accessibility data to predict thousands of single-cell 3D chromatin conformations within minutes, dramatically outpacing traditional experimental approaches [89]. AlphaGenome takes 1 Mb DNA sequences as input and predicts thousands of functional genomics tracks at single-base resolution across multiple modalities. These modalities include gene expression, transcription initiation, chromatin accessibility, histone modifications, transcription factor binding, chromatin interaction maps, splice site usage, and the coordinates and strength of splice junctions [90].

## Therapeutic strategies targeting 3D genome in cancer

The therapeutic landscape of targeting 3D chromatin architecture is rapidly evolving, with multiple classes of agents demonstrating the capacity to reshape genome topology through distinct molecular mechanisms. Epigenetic modifiers represent the most established approach, wherein HDAC inhibitor vorinostat promotes histone hyperacetylation to relax chromatin compaction and alter chromatin interactions [91], while DNA methyltransferase inhibitors including decitabine, azacitidine, and 5-AZA reverse aberrant DNA methylation patterns, thereby restoring normal chromatin communication and TAD boundaries that are frequently disrupted in malignancy [11, 92, 93]. Similarly, EZH2-targeted therapeutics, including the EZH2 PROTAC degrader MS177 and small-molecule EZH2 inhibitors such as GSK126, C36, and tazemetostat, attenuate H3K27me3-mediated gene silencing and consequent chromatin condensation, indirectly facilitating the reorganization of oncogenic 3D genome configurations toward a more normalized state [94–97].

Beyond these epigenetic-centric modalities, emerging strategies directly interfering with architectural proteins and phase-separated nuclear compartments are gaining substantial momentum. Curaxin (CBL0137) operates through a unique mechanism by intercalating into DNA and inducing the dissociation of CTCF from its cognate binding sites, thereby disrupting chromatin looping and suppressing pathological enhancer-promoter interactions that drive oncogene expression [98]. The BRD4 PROTAC degrader ARV-771 achieves potent destruction of super-enhancer hubs, highly concentrated clusters of transcriptional machinery, by eliminating BRD4-mediated phase separation and chromatin recruitment, effectively dismantling the structural scaffolds sustaining malignant transcriptional programs [99–101]. KPT-6566 targets the cohesin regulatory network by specifically disrupting the interaction between STAG1/STAG2 and SCC1 as well as their association with double-stranded DNA, compromising cohesin-mediated loop extrusion and sister chromatid cohesion essential for proper chromosome architecture [102]. Furthermore, Nexturastat A, a phospho-HDAC6 liquid-liquid phase separation inhibitor, suppresses tumor progression through mechanisms of chromatin remodeling and transcriptional reprogramming, highlighting the therapeutic potential of modulating biomolecular condensates that increasingly recognized as critical organizers of nuclear genome topology [103]. Collectively, these agents illustrate the expanding repertoire of pharmacological tools capable of manipulating 3D chromatin architecture, from indirect epigenetic modulation to direct perturbation of structural proteins and phase-separated domains, offering promising avenues for precision cancer therapy.

## Conclusions

The 3D organization of chromatin represents a fundamental regulatory layer that is profoundly disrupted in cancer. As reviewed herein, neoplastic transformation is accompanied by extensive architectural rewiring across multiple hierarchical scales, from global A/B compartment switching and TAD boundary erosion to the aberrant formation or dissolution of enhancer-promoter loops. These structural alterations are not merely epiphenomena of malignant transformation but constitute functional drivers of oncogenic transcriptional programs, tumor heterogeneity, metastasis and therapeutic resistance.

The multifactorial etiology of 3D chromatin dysregulation in cancer underscores the complexity of this regulatory landscape. Structural variants can directly reshape topological domains by deleting or duplicating boundary elements, while mutations in CTCF and cohesin subunits compromise the integrity of insulating neighborhoods and loop extrusion processes. Epigenetic mechanisms, including DNA methylation, histone modifications, and the activity of chromatin remodeling complexes, further modulate the accessibility and stability of architectural protein binding sites. Emerging evidence also implicates transcription factors as pioneering forces that remodel local chromatin topology, liquid-liquid phase separation as a biophysical principle underlying condensate-mediated chromatin reorganization, and non-coding RNAs as scaffolds or decoys in 3D organization. Viral integration exploits host chromatin architecture to access enhancer landscapes, illustrating how exogenous insults can hijack endogenous spatial genome regulation.

Technological innovations have been instrumental in advancing this field. From the foundational Hi-C methodologies to high-resolution Micro-C, single-cell 3D genome mapping, and integrative multi-omics approaches, these tools have progressively unveiled the cancer genome’s spatial complexity with increasing precision. The advent of CRISPR-based architectural editing, live-cell imaging of chromatin dynamics, and computational frameworks integrating 3D structure with epigenomic and transcriptomic data now enables mechanistic interrogation and predictive modeling of structural aberrations.

Therapeutic targeting of 3D chromatin architecture spans from established epigenetic modifiers to emerging agents that directly perturb architectural proteins and biomolecular condensates, collectively expanding the pharmacological toolkit for precision cancer intervention.

Looking forward, several critical challenges and opportunities merit attention. First, the functional causality of specific 3D structural changes remains difficult to establish; distinguishing driver architectural alterations from passenger events will require sophisticated genetic perturbation studies in isogenic models. Second, the therapeutic translational potential of targeting 3D genome regulators, such as CTCF, cohesin, and chromatin remodelers, must be carefully weighed against the risk of disrupting essential architectural functions in normal tissues. Third, the integration of 3D genomic information into precision oncology frameworks holds promise for biomarker development and patient stratification, particularly in predicting drug response and resistance mechanisms. Finally, the convergence of 3D genome biology with real-time spatial transcriptomics and artificial intelligence-driven modeling will likely yield a predictive, dynamic understanding of nuclear organization, revealing therapeutic vulnerabilities that transcend conventional static, target-centric paradigms.

In summary, the study of 3D chromatin architecture in cancer has transitioned from descriptive mapping to mechanistic understanding, and is poised to enter an era of clinical translation. As our comprehension of the spatial genome deepens, so too does the prospect of exploiting these insights for the diagnosis, prognostication, and rational treatment of human malignancies.

## Data Availability

No datasets were generated or analysed during the current study.

## Abbreviations

3D: Three-dimensional
AI: Artificial intelligence
AML: Acute myeloid leukemia
BCL: B-cell lymphoma
CTCF: CCCTC-binding factor
DLBCL: Diffuse large B-cell lymphoma
ER: Estrogen receptor
GBM: Glioblastoma
SVs: Structural variations
T-ALL: T-cell acute lymphoblastic leukemia
TADs: Topologically associating domains
